# Conceptualisation of an Efficient Particle-Based Simulation of a Twin-Screw Granulator

**DOI:** 10.3390/pharmaceutics13122136

**Published:** 2021-12-12

**Authors:** John P. Morrissey, Kevin J. Hanley, Jin Y. Ooi

**Affiliations:** School of Engineering, Institute for Infrastructure & Environment, The University of Edinburgh, Edinburgh EH9 3JL, UK; J.Morrissey@ed.ac.uk (J.P.M.); K.Hanley@ed.ac.uk (K.J.H.)

**Keywords:** powder agglomeration, Discrete Element Method, cohesion, wet granulation, twin-screw granulation

## Abstract

Discrete Element Method (DEM) simulations have the potential to provide particle-scale understanding of twin-screw granulators. This is difficult to obtain experimentally because of the closed, tightly confined geometry. An essential prerequisite for successful DEM modelling of a twin-screw granulator is making the simulations tractable, i.e., reducing the significant computational cost while retaining the key physics. Four methods are evaluated in this paper to achieve this goal: (i) develop reduced-scale periodic simulations to reduce the number of particles; (ii) further reduce this number by scaling particle sizes appropriately; (iii) adopt an adhesive, elasto-plastic contact model to capture the effect of the liquid binder rather than fluid coupling; (iv) identify the subset of model parameters that are influential for calibration. All DEM simulations considered a GEA ConsiGma™ 1 twin-screw granulator with a 60° rearward configuration for kneading elements. Periodic simulations yielded similar results to a full-scale simulation at significantly reduced computational cost. If the level of cohesion in the contact model is calibrated using laboratory testing, valid results can be obtained without fluid coupling. Friction between granules and the internal surfaces of the granulator is a very influential parameter because the response of this system is dominated by interactions with the geometry.

## 1. Introduction

Wet granulation is a process used to create larger stable agglomerates (granules) from fine powders. This has many desirable outcomes such as improving flowability, compactibility and homogeneity. Granulation is commonly employed in the food, pharmaceutical, detergent and fertilizer industries. Despite its widespread adoption, it is often inefficiently operated [1,2], with high recycle ratios in continuous processes and high rejection rates in batch processes [3]. Wet granulation is the most common type of granulation and, in pharmaceutical applications, it is a critical step in tablet manufacturing that affects the uniformity and compactibility of the final dosage form.

Traditionally, batch granulation was the favoured granulation approach in the pharmaceutical industry due to the challenges associated with continuous processing such as the changeover cost and inability to monitor product quality reliably. Since the introduction of Quality by Design (QbD) and Process Analytical Technology (PAT) by the FDA in 2003 [4] and due to recent advances in continuous monitoring, there has been a move from traditional batch towards continuous processing. Although tremendous efforts have been made to gain scientific insight into the granulation process [5,6,7,8,9,10,11,12], a fundamental understanding of wet granulation is still lacking due to the complexity of the mechanisms involved, i.e., governing rate processes of wetting and nucleation, aggregation and consolidation (or consolidation and coalescence), breakage and attrition, and layering [1,9]. 

Twin-screw granulation developed from early work on single- [13] and twin-screw extruders [14] in the late 1980s. It has become popular within the pharmaceutical industry in the past decade due to the many advantages it offers over high shear and fluidised bed granulators including limited or no scale-up requirement, lower space requirements, continuous operation with monitoring and higher throughput. Since a patent was awarded to Ghebre-Sellassie et al. [15], there has been increased adoption of this method of granulation in industry.

A twin-screw granulator (TSG) consists of three main components: the powder feed, liquid addition mechanism and two intermeshed screws, which may be either co- or counter-rotating, enclosed in a barrel. The powder is fed into the screw barrels at one end and exits the open end of the barrel. This is in contrast to twin-screw extruders where the granulated product is forced through a die or plate at the end of the barrel. Twin-screw granulators are often described by their length-to-diameter (L/D) ratio [16] with extruder lengths in the range of 12–50 L/D common in industry [17]. Screws are required to tightly fit within the bore of the barrel to create a closely confined flow path for materials. Researchers have reported that the confinement offered by the low clearance between the screws and barrel ensures a similar shear history for all particles, which helps produce more consistent granules than batch granulation processes [17,18]. Screws typically operate part-filled, with solid fraction dependent on screw geometry, material feed rate and screw speed [16]. The screws are modular in nature and comprise elements of different types to give the required configuration.

In general, there are four element types: conveying elements, kneading elements, chopping elements and comb mixer elements (not considered in this paper). Multiple elements are combined to form a block; multiple alternating blocks form the complete screw. Kneading blocks impart high mechanical energy to the wetted material, producing high shear forces, compaction and distributive mixing. Further details on screw elements, profiles and configuration can be found in the literature [16,17,18,19,20,21,22].

Depending on the angle of offset, kneading blocks can produce forwarding or reversing flow [23,24]. Reversing kneading blocks force material back against the direction of flow, which leads to areas of high pressure and compaction. Although this allows strong granules to be formed, there is a high likelihood of blockages [25]. Chopping elements are shortened kneading elements whose purpose is to break up oversized agglomerates at the end of the screw.

Twin-screw granulation has been studied in much detail in recent years [16,17]. Several experimental studies have investigated the effects of key process variables [26,27,28,29,30,31,32,33,34,35,36,37], screw configurations [24,25,38,39,40,41,42] and formulation variables [26,36,40], and have resulted in a regime map [43] of the twin-screw granulator. However, these experimental studies cannot provide any insight into the micro-scale phenomena that govern granulation processes. Particle-scale simulations have the potential to provide this insight. The Discrete Element Method (DEM) has become the dominant particle-scale simulation tool in the last 20 years. DEM has been applied to investigate various wet granulation processes, e.g., Mishra et al. [44] and Liu et al. [45] investigated granulation in a rotating drum; Goldschmidt et al. [46] and Kafui et al. [47,48] simulated fluidised bed granulation; Gantt and Gatzke [49], Hassanpour et al. [50], Nakamura et al. [51], Watson et al. [52], Tamrakar et al. [53] and Börner et al. [54] studied high shear granulation. Researchers have also explored granulation processes by coupling DEM and population balance modelling [55,56,57,58,59]. However, there are few prior DEM studies of twin-screw granulation. Dhenge et al. [29] studied the effect of binder amount and binder viscosity at varying powder feed rates on the granulation behaviour and final granule properties. Due to the large computational cost, their simulations use conveying screws only within a 16 mm periodic domain. They also do not include cohesion between particles in the simulations. Only a qualitative visual comparison was made to the experimental results. Recently Zheng et al. [60] have used DEM to study residence time distributions for dry, cohesionless, mono-sized elastic spheres of various sizes for a fixed configuration of conveying elements with two blocks of kneading elements at various operating RPMs. Zheng et al. [61] extended the study further to include the effect of particle shape in the TSG. Spherical particles were observed to have slightly lower mean residence times than particles of other shapes, which were largely the same, regardless of shape. The influence of the many DEM parameters on the residence time has not been investigated in these studies, which only considered dry cohesionless materials. Kumar et al. [62] used mono-disperse periodic DEM simulations to study liquid distributions within the mixing zone of a GEA ConsiGma 25 TSG; however, this zone was only one kneading element thick (8× particle diameters). While liquid transfer and associated agglomeration could be observed, the limited domain means no longitudinal travel or transfer from conveying elements were considered in this model, providing a somewhat isolated overview of the dynamics within a TSG.

The objectives of this research study are threefold:

- To develop and verify computationally efficient reduced-scale DEM models that closely match full-scale simulations of the entire granulator;
- To carry out a comprehensive sensitivity study to explore how operating parameters and DEM model parameters affect the performance of a TSG, and thereby identify the subset of influential parameters requiring calibration (noting that this study does not attempt to perform a full calibration of the granular material against a real solid);
- To demonstrate that the effect of liquid binder can be captured using an adhesive, elasto-plastic contact model.

In order to create a numerical model that is capable of producing numerical predictions that can be validated against experimental results, it is necessary to first verify that the conceptual model is operating in the correct manner, similar to its real-life counterpart. As such, the operation of the TSG model is verified by comparing the particle dynamics, flow characteristics and residence time distributions against previously reported sources. Validation is concerned with establishing the predictive capability of the computational model in relation to the real system. Computational results should be compared with independent experimental results, and a computational model will be considered validated once the discrepancy between the simulation and experimental result of interest falls within pre-determined bounds. This discrepancy must be evaluated against the degree of uncertainty in both the validation experiment and the multiscale model. Validation of the model outputs against careful experimental measurements such as granule porosities and granule size distributions is not part of this study.

## 2. Twin-Screw Granulator Model

**Figure 1:**
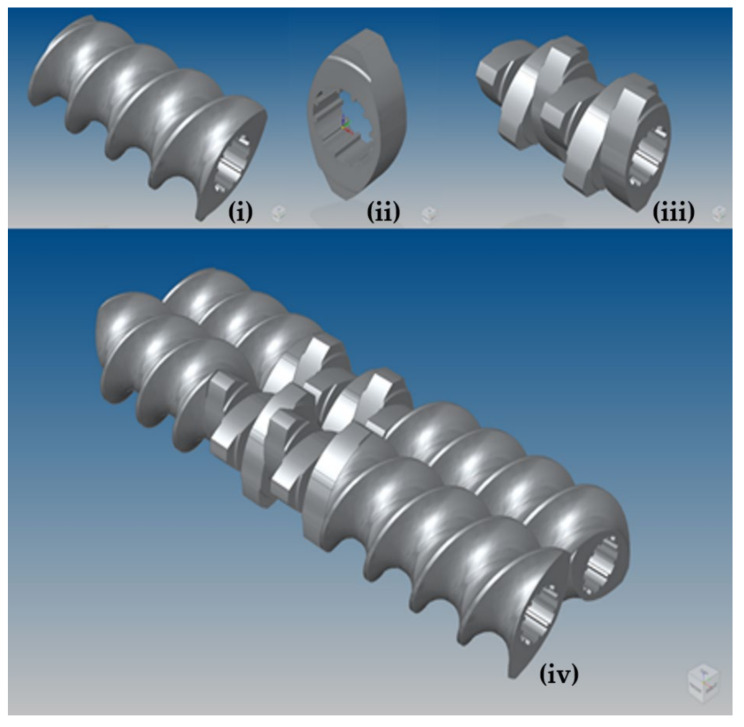
CAD model of ConsiGma 1 screw. Top row, from left to right: (i) Conveying element of length 37.5 mm (ii) Kneading element (iii) 6 × 60 R block of kneading elements; Bottom row: (iv) 6 × 60 R block of kneading elements with adjoining conveying elements.

The test apparatus chosen is a ConsiGma™ 1/25 continuous tableting line (CTL) manufactured by GEA. The ConsiGma 1/25 granulator consists of two co-rotating screws, each housed within a 25 mm diameter barrel. The dimensions of the TSG and screws are summarised in Table 1 and Table 2, respectively. A key figure is the free barrel fraction of 0.4731, i.e., around 47% of the total volume of the granulator barrels is free space for particles to occupy. The ConsiGma 1/25 granulator uses three types of screw element: conveying, kneading and chopping. CAD models of these individual elements were constructed in 3D, as shown in Figure 1. All elements consist of the same cross-section. Conveying elements of three different lengths were formed with a helical extrusion, and kneading and chopping elements with simple extrusions. The key difference between kneading and chopping elements is the depth, with kneading elements being 6.25 mm deep and chopping elements being just 4.167 mm deep.

### 2.1. DEM Model Configuration

**Figure 2:**
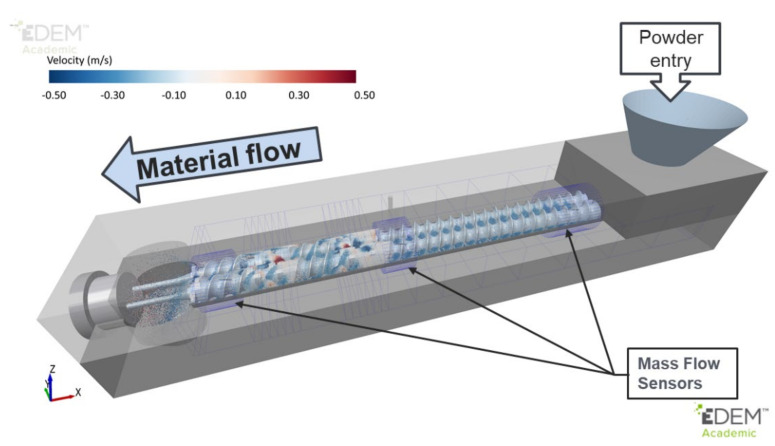
Full−scale DEM model of ConsiGma 1/25.

DEM simulations of the ConsiGma TSG were carried out with the commercial code EDEM [63]. The CAD model described in the previous section was imported into EDEM (Figure 2). A high level of precision was used during import to ensure that no unnatural edges existed in the intermeshing region where screw clearances are very tight. Twin-screw granulators are operated at steady state: the total mass entering the system is equal to the mass leaving the system. In the simulations, three mass flow sensors were included along the barrel to assess when the granulator reached a steady state during operation.

**Figure 3:**
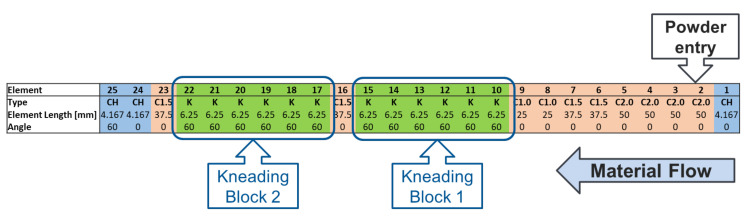
Typical experimental screw configuration, as used in the full-scale DEM simulations. CH represents a chopping element, C1.5 represents a conveying element of length 1.5 times the screw lead and K denotes a kneading element.

The stainless-steel barrel and screws were modelled as rigid bodies in EDEM. Appropriate rotational dynamics were added for the chosen screw speed. The screw configuration used in the simulations is that of a typical experimental setup, summarised in Figure 3, which consists of two blocks of six kneading elements with a 60° offset in a reversing configuration, separated by a 1.5D conveying element. Two chopping elements were located at the screw exit to break up any oversized agglomerates that may have formed.

Both cohesionless and cohesive systems were studied. Cohesionless systems are first introduced to study the effect of parameters such as friction and restitution, in recognition of the fact that most DEM simulations of twin-screw granulators still adopt simple cohesionless models. The subsequent inclusion of cohesion allows its influence to be distinguished from the other parameters.

**Figure 4:**
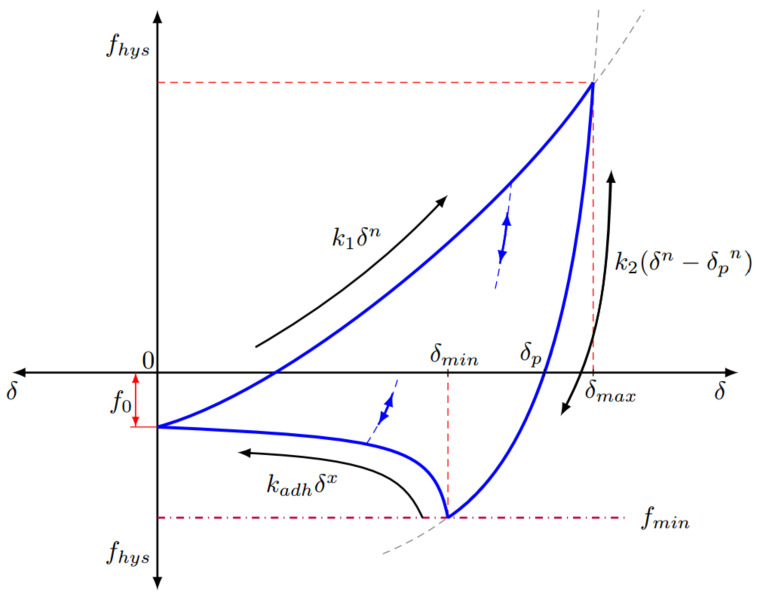
Force–displacement behaviour of the Edinburgh Elasto-Plastic Adhesion model.

In the absence of cohesion, a conventional Hertz-Mindlin contact model was used. For cohesive interparticle interactions, the Edinburgh Elasto-Plastic Adhesion (EEPA) model [64,65] (Figure 4) was used: an adhesive, elasto-plastic contact model that captures the key characteristic behaviour for agglomerates. This non-linear model is used to capture the role of the liquid binder in the TSG. It is based on the physical phenomena observed in adhesive contact between micron-sized particles or small agglomerates [66]. The EEPA model accounts for both the elastic–plastic contact deformation and the contact-area-dependent adhesion. Key parameters of this model are the constant pull-off force (*f*_0_), the slope exponent (*n*) and the contact plasticity ratio, which relates the unloading/reloading stiffness *k_2_* to the loading stiffness *k_1_*. The EEPA model has been successfully used to model the behaviour of wet iron ore fines [64,67,68], detergent powders [69,70], powder mixing [71] and cohesive soils [72]. The self-cleaning nature of intermeshing, co-rotating screws means that the cohesionless Hertz-Mindlin contact model was used for all particle–geometry contacts. 

Breakage of the primary particles was not considered in the model. However, agglomerates formed by cohesion were able to break and reform, similar to real agglomeration behaviour. Liquid migration was not considered in the model to reduce the computational cost. Values of key input parameters used for the cohesionless simulations are given in Table 3. These parameters form the reference case for the sensitivity study presented later. The chosen bulk density is that of a typical pharmaceutical solid: paracetamol. The remaining parameters are chosen as typical median values that are representative of a cohesive powder. The “standard deviation” of 0.05 in Table 3 defines the width of the normal Gaussian distribution in particle diameter, about a mean value of 400 μm. This distribution is truncated at an upper and lower limit (0.5*x*–1.5*x*), relative to the mean. The effect of cohesion in the TSG is considered through a set of cohesion parameters that result in high levels of cohesion (given later in Table 4). These parameters have not been calibrated for any specific material or level of liquid binder and are simply to explore the effect of cohesion in the system and its relative importance.

The main processing parameters in twin-screw granulation include the liquid-to-solid (L/S) ratio, the feed rate, the screw speed, the screw configuration and the barrel temperature. The combination of screw speed and feed rate leads to the development of a specific fill level or porosity on each element type, with a lower porosity typically found on kneading elements. The stress state developed is largely determined by the screw speed in combination with the geometric clearances in relation to particle size. A higher powder feed rate increases the degree of compaction and densification of the powder in the TSG barrel. The feed rate chosen for the simulations is a typical value of approximately 14.4 kg/h. A single screw speed of 600 RPM was chosen. The level of cohesion in the simulations was used to capture the effect of liquid in the system. The level of cohesion could be calibrated against flowability measurements from experimental characterisation tests, e.g., direct shear or uniaxial tests, for the various L/S ratios used. Thermal effects and barrel temperature were not considered in these DEM simulations.

### 2.2. Numerical Instabilities

There are various instabilities that can develop in a numerical model, which require careful consideration to avoid unwanted effects on the simulation results. Numerical instabilities in DEM simulations can arise from various sources, but the most common source of errors is the integration timestep. The sensitivity of the results to the integration timestep was checked independently for 1%, 10% and 20% of the critical Rayleigh timestep and no significant variation in results was noted. As such, all simulations were carried out with a timestep of 5 × 10^−7^ s, which is 10% of the critical Rayleigh timestep for 400 µm particles or 20% for 200 µm particles. This timestep is sufficiently low to ensure that the contacts are identified accurately in this highly dynamic environment. Although a reduced shear modulus has been chosen to reduce the computational time, it is kept large enough to ensure numerical accuracy [73,74].

Given the tight tolerances at play in a twin-screw granulator and the generally large L/D ratios for the barrel, the accuracy of geometry discretization as well as the choice of elements is also of importance here. The curvature of the screw and barrel needs to be sufficiently captured to ensure particles are not getting “pinched” in gaps that are artefacts of the discretistation of the CAD geometry. It is also important to ensure that small, well-shaped meshes are used and to avoid any stretched triangles, especially in the barrel, as this could cause some strange effects.

Finally, adding large amounts of cohesion to the system can cause many problems such as creating excessive overlaps at collisions to forming a single agglomerate containing all particles in the simulation. It should also be noted that even if the amount of cohesion used is not excessive, it can still lead to the build-up of material in the kneading elements that would cause jamming of the real granulator. In this numerical situation, rather than the screws stopping, the particles will just be forced through the barrel walls. 

### 2.3. Full-Scale vs. Reduced Domain Models

In this study a comparison is made between a full-sized domain model of the granulator and a more computationally efficient reduced domain model to investigate whether it is possible to use computationally efficient models without affecting the correctness and reliability of the simulation results.

#### 2.3.1. Full-Size Computational Domain

This simulation captures the continuous flow through the granulator where transitions between different element types lead to different phenomena. The full length of the TSG has been modelled from inlet feed to outlet. It was observed that due to the consistent behaviour of particles on conveying elements (which serve only a transport purpose before liquid injection), the particle generation point could be moved to mid-way along the granulator to element number 7 on Figure 3 (immediately before the liquid inlet points on the physical TSG) without any observable difference in results. The computational cost of simulating all of these elements is high and moving the particle generation location reduced the required run-time by approximately 40%. It is important to note that the measured residence time for the shortened granulator would need to be adjusted for experimental comparisons where the granulator length cannot be reduced.

#### 2.3.2. Reduced Domain Models

The computational cost of running the full simulations is considerable: from start-up to steady state, and then operation at steady state for several seconds, requires millions of particles over a typical 10–20 s simulation. To reduce this cost, the twin-screw granulator can be broken down into different sub-domains of interest, such as a block of conveying elements, a block of kneading elements or a block of conveying and kneading elements.

A reduced domain model can be created to focus on the specific sub-domain of interest and can be created with either periodic boundaries at each end of the domain in the direction of flow (termed *periodic*) or with particle generation at one end of the screw and the standard domain from which particles will exit at the other (termed *non-periodic*). These reduced domain models are numerically efficient as the number of particles is considerably reduced. This can allow greater fidelity in the models through the use of smaller particles or the implementation of more complex physics for the same computational cost as the full-size granulator. Selecting the elements for the reduced domain model requires consideration of the key zones in the full granulator. The mixing zones containing the kneading elements are significantly more important to the granulation process than the conveying elements. As such, these mixing zones are the focus of the reduced models in this study.

At this point it is worth considering some of the limitations of the two types of reduced domain models. A periodic sub-domain model cannot consist only of kneading elements since these elements are mixing elements rather than transport elements, and without sufficient transport elements there would be little or no forward flow in the periodic sub-domain model. This would lead to residence times that would tend towards infinity as there is no longitudinal flow being provided. The length of the periodic sub-domain model must be such that that the same screw position is matched on the start and the end screw profiles to prevent loss of particles and ensure a clean and error-free recycling of the particles. This can make periodic sub-domain models longer than non-periodic sub-domain models, which do not need to recycle particles. Periodic sub-domain models will have a fixed number of particles, which means they cannot enforce a fixed flow rate, whereas non-periodic sub-domain models utilise dynamic particle generation and can be used to study the granular behaviour under fixed flow rates. Periodic sub-domain models cannot be used to study the developing granule size distribution as formed granules would be recycled through the initial flow boundary instead of virgin feed. In this type of study some models with continuous generation would be required.

#### 2.3.3. Implemented Reduced Domain Model

**Figure 5:**
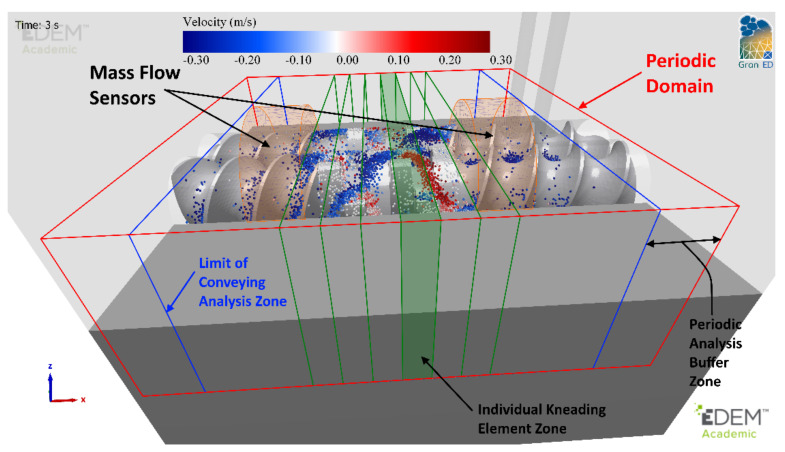
Periodic simulation zones at steady state.

An equivalent periodic model that comprises a full block of kneading elements (six elements) and the adjoining conveying elements was developed to investigate the complex behaviour within a kneading block. This is shown in Figure 5. The extent of the simulation domain, which is periodic in the x-direction, is outlined in red. The kneading elements in the centre of the system are outlined in green, with one kneading element zone, which is the same thickness as a kneading element, highlighted. The extent of the adjoining conveying elements for analysis purposes is marked by the blue zones. The difference between the simulation domain (red) and the analysis domain (blue) creates buffer zones where the particles are not considered for analysis, as they may be affected by some backflow or particles oscillating at the periodic boundary. The average mass of particles from the full-sized simulation, for the full duration at steady state in the same domain, was generated statically in the free space around the screws as the starting point for the periodic system. The simulation was then run for a short period of time to reach a steady state—approximately the time taken for a single particle to circulate through the system (0.5–0.75 s)—to allow particles to distribute naturally along the elements. All data analysis was carried out after this initial start-up time. This setup produces a very good qualitative match to the flow patterns observed in the full-scale simulations.

#### 2.3.4. Comparison to Full-Scale Simulation

**Figure 6:**
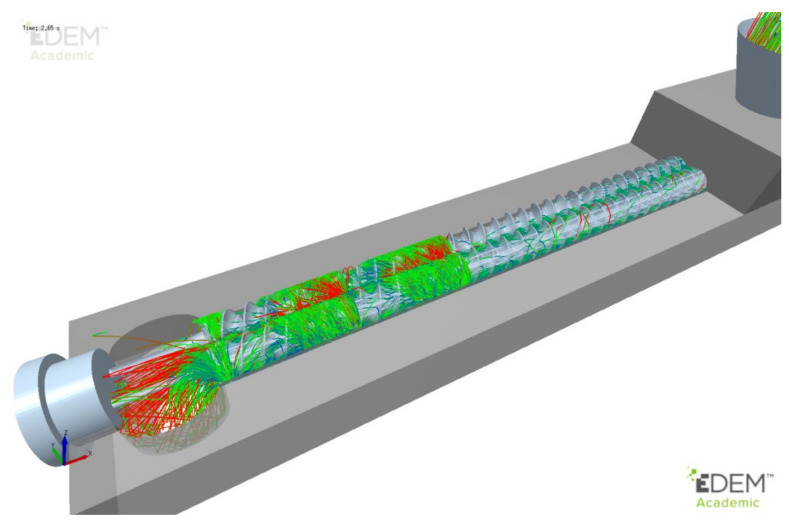
Particle streamlines for full granulator.

The full-scale DEM simulation was run for 2 s after steady state operation had been achieved. Particle streamlines were extracted for a random selection of particles and are shown in Figure 6. These streamlines and resulting residence times correlate well with those measured experimentally using PEPT (positron emission particle tracking) [7,75].

**Figure 7:**
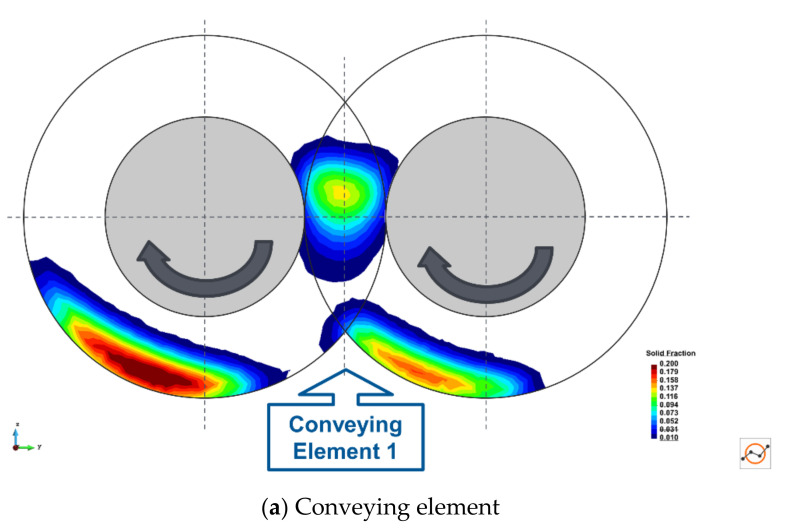

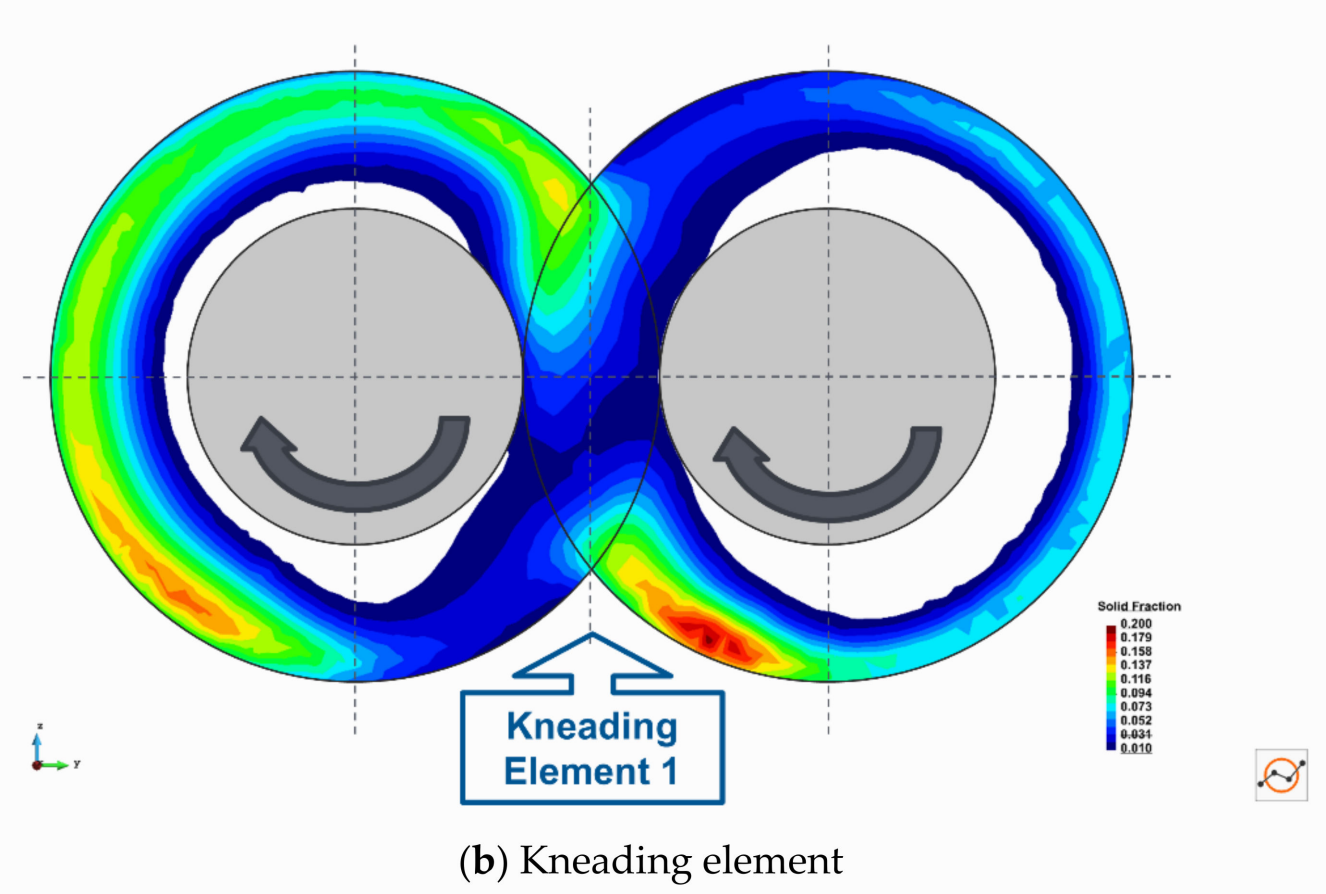
Transport locations in different element types.

Cross-sections of the temporally (5 screw revolutions) and spatially averaged solid fraction are shown in Figure 7 for the two main element types: conveying and kneading elements. The figure shows that there are three main transport locations for the conveying element: between the screws in the intermeshing zone and at the bottom of each screw bore. The kneading elements show a different pattern due to their flat nature and tend to only push particles around the plane of the element. The DEM simulations predict more material in one bore of the conveying elements, where the driving screw is located, than the other. This matches previous experimental observations [76].

**Figure 8:**
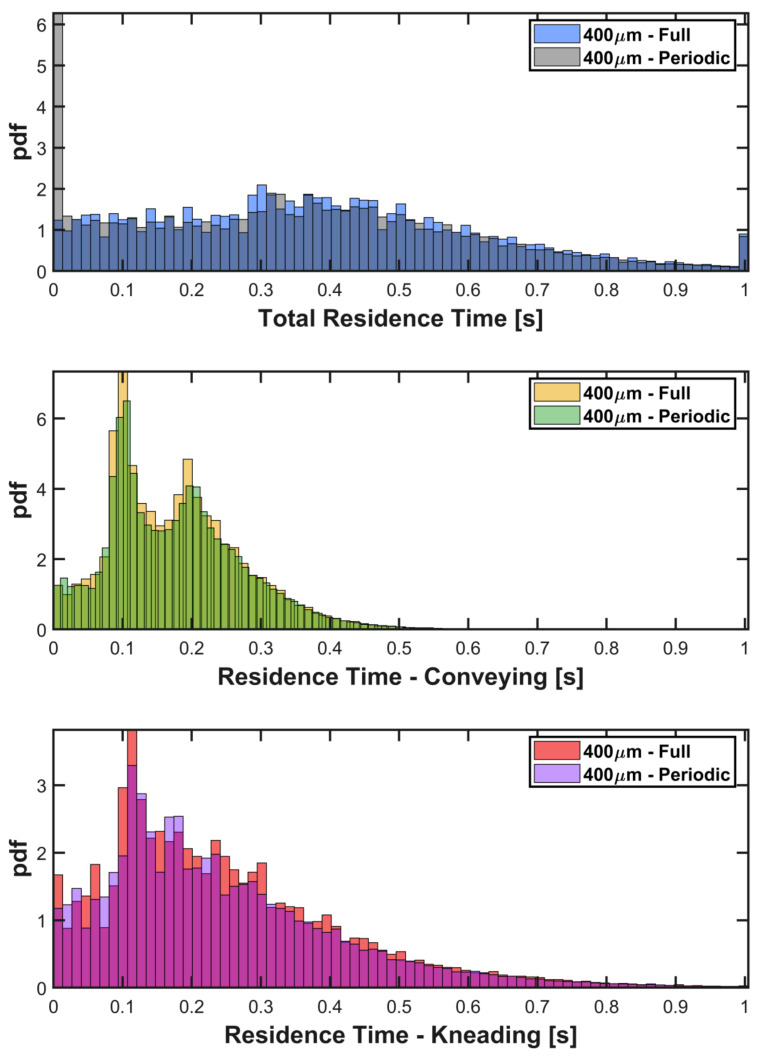
Comparison of the Residence Time Distributions (RTDs) for the full model and periodic simulation.

The Residence Time Distribution (RTD) for the full TSG simulation was extracted, which shows a mean total residence time of approximately 3 s. This is in the region of what has been measured experimentally for a free-flowing material in similar equipment [7,27,28,39]. RTDs for the full-size DEM simulation and the periodic simulation in the same domain, over a 1 s period at steady state, are compared in Figure 8. The periodic simulation is capturing the same behaviour as the full-size simulation. For the total residence time, the periodic results include a spike at 0 s, which is an artefact due to the re-circulation of particles through the periodic domain and the time they spend in the buffer zone at each end. The agreement is also excellent for the RTDs by element type.

**Figure 9:**
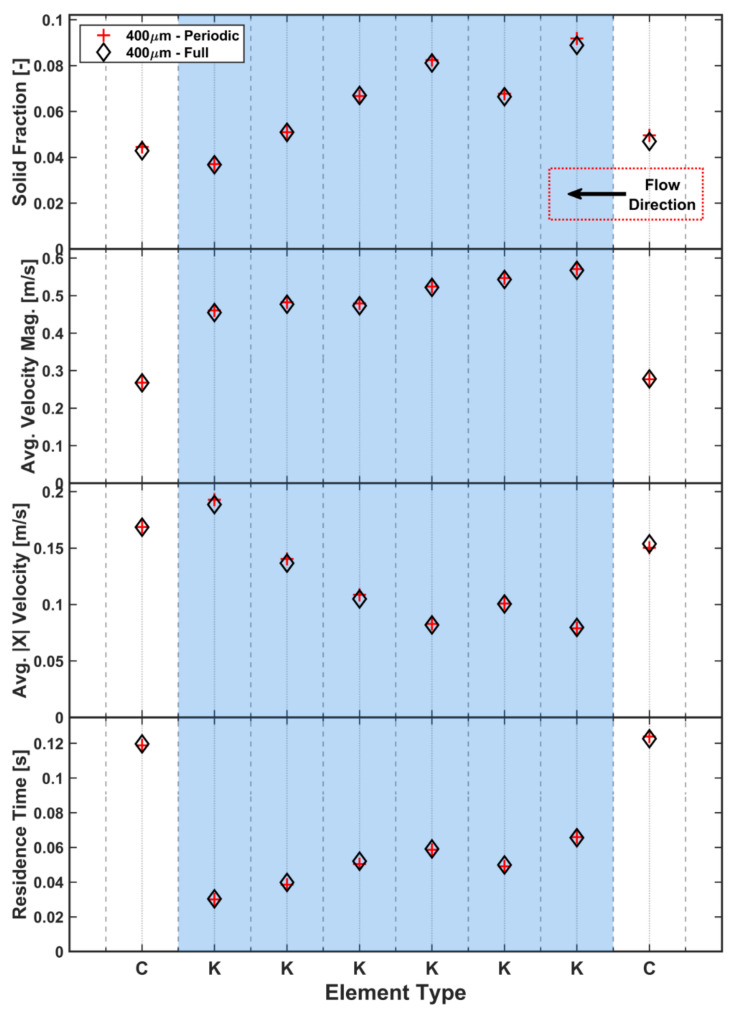
Comparison between periodic and full-scale simulations for various measured quantities.

Figure 9 shows a comparison between various temporally-averaged measured quantities (residence time, solid fraction, average velocity magnitude and average longitudinal (X) velocity) per element for the two model types. The results from both model types are in excellent agreement, verifying that the periodic system is capable of reproducing the same phenomena observed in the full-size simulation and can safely be used in the sensitivity study to investigate the effect of the various DEM parameters. Both models show that there is a strong link between the residence time and solid fraction for each element as the largest residence time per unit length is found where the highest solid fractions exist. It should be noted that the measured region of the conveying elements is longer (approx. 3.5*x*) than the kneading elements. Figure 9 also shows the velocity magnitude and longitudinal velocity comparisons for the two models. The axial velocities show a strong connection to the respective solid fractions and residence time values, whereas this is less clear for the velocity magnitudes, which are similar across all kneading elements. The velocity magnitude, resulting from the longitudinal and in-plane movement, is, however, much higher for kneading elements than for conveying elements due to the rotational path enforced by the geometry and is, therefore, heavily influenced by the screw RPM. 

### 2.4. Influence of Particle Size

**Figure 10:**
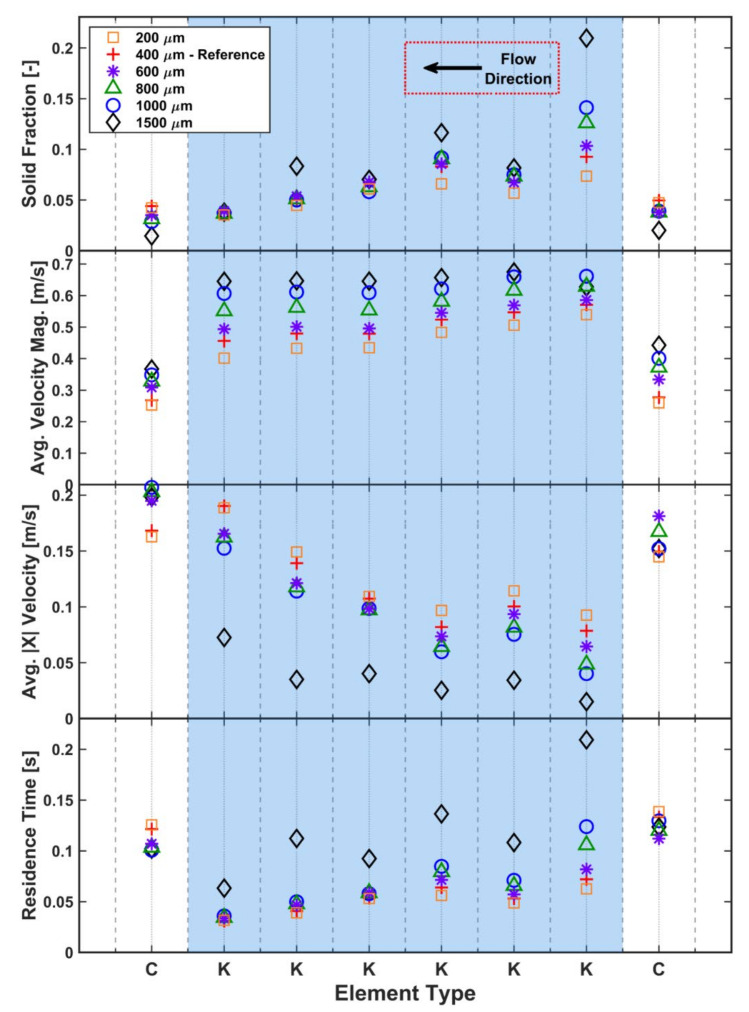
Effect of particle size on solid fraction, velocity and residence time.

**Figure 11:**
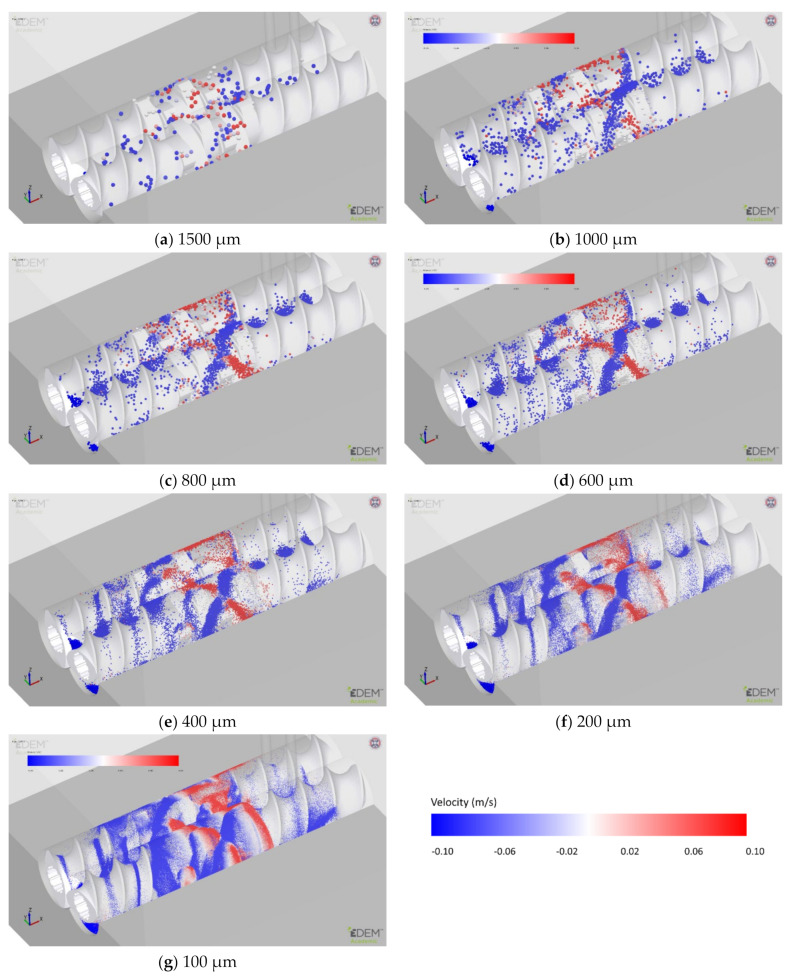
Particle snapshots at steady state as the mean particle diameter is varied. The particle colour indicates longitudinal velocity in the direction parallel to the screws; a negative velocity indicates the movement of particles towards the outlet of the granulator.

The mean particle diameter was varied from a reference value of 400 µm to values in the inclusive range between 200 µm and 1500 µm, all with the same truncated normal size distribution with the same standard deviation. The total mass of particles generated in the domain is the same for all particle sizes, being obtained from the steady-state mass in the same elements in the full-size model. The effects of the change in mean particle size on the solid fraction, velocity and residence time are shown in Figure 10. Figure 11 shows the snapshots at the same steady-state time instant for all simulations.

The solid fraction results on conveying elements fall into three separate classes for small (<500 µm), medium (500–1000 µm) and large (>1000 µm) particles. Large particles lead to the lowest solid fraction on conveying elements due to the geometric constraints of the screw geometry: larger spheres cannot occupy the space around the screws as efficiently as smaller spheres. The kneading block of six elements appears to act as two blocks of three elements, with significantly different behaviours observed on the first three and the last three kneading elements. In the last three elements, the observed solid fractions are largely the same for all particle sizes, apart from the large 1500 µm particles. The first three kneading elements show significant variation with the larger particles generally having a higher solid fraction.

For the longitudinal (X) velocity of the particles along the barrel, the largest particles have the lowest longitudinal velocity on the kneading elements, suggesting that these particles experience more obstructions by geometrical constraints at the transitions between elements, hampering their path through the granulator. All particle sizes follow the same general trend where there is a significant velocity decrease at the transfer from conveying to kneading elements. However, the magnitude of the retardation is heavily dependent on particle size, with the larger particles reduced to the lowest velocity. The 1500 µm particles do not follow the general trend of significantly increasing longitudinal velocity as the particles progress through the kneading block, with the velocity remaining almost constant until the last kneading element before an increase is noted. All other sizes show a clearer velocity increase along the kneading block and have returned to the original screw translational velocity by the time they have left the kneading block.

Figure 10 also shows a consistent trend of increasing velocity magnitude with increasing size, with an almost constant value for each element type. Interestingly, there is a large variation in the velocity magnitudes for differently sized particles in the conveying elements, with larger particles having larger velocities. This appears to be a result of the number of particles in the system. As the size of the particles increases, the number of particles in the system decreases, which can lead to decreasing levels of particle–particle collisions in the system. With only several large particles able to sit on a conveying element at once, there are relatively few interparticle contacts, leading to lower energy dissipation. This results in the larger particles being more active in the screw elements.

A similar trend of high velocity magnitude for larger particles is also observed in the kneading elements. Within the kneading zone, the increased velocities can also be attributed to the reduction in particle numbers. In the kneading zone, particle collisions play a significant role in helping particles progress along the screw. Without the particle collisions to help “nudge” particles onto the next element, the larger particles have a tendency to remain circulating in-plane, being pushed around by the flat lobes of the kneading elements. There is also a geometric constraint at play in the kneading zone, with the larger particles more likely to collide with the next kneading element and remain on the current element than smaller particles, which are less likely to be constrained in this manner. These factors contribute to the reduced longitudinal velocity in the larger particles, which is also reflected in the observed residence times that are highest for the largest particles.

Pradhan et al. [77] found from experiments and geometrical analysis on static 3D CAD models that particle breakage was related to the maximum void space on an element, with 20% of particles on mixing elements broken at just 60% of the maximum size and all particles suffering some breakage at less than the maximum size. This geometrical breakage constraint was recently implemented in a new population balance model kernel for particle breakage [78] and should be considered when choosing particle sizes for simulations so as to not adversely affect the simulation results. The simulation results in the current study suggest the existence of a critical ratio of particle diameter to the screw void space (effectively the difference between the screw’s root and outer radii) for unhindered particle flow that is significantly less than the breakage limit identified by Pradhan et al. [77]. For the configuration used in this study, the maximum dimensions within the void spaces are approximately 5.5 mm and 6.25 mm for conveying and kneading elements, respectively, suggesting a diameter-to-void limit of approximately 10–20% for unhindered particle flow. This limit may also be affected by both the offset angle and direction of the kneading/mixing elements, which could reduce the maximum dimensions within the void spaces below the figures quoted here.

The significance of the particle size in relation to the screw–barrel clearance can also be observed in Figure 10. This critical clearance is 440 µm (Table 1 and Table 2) in this model, which means approximately half of the particle sizes studied will not pass through this outer region. With the exception of kneading element 1, the particle behaviour that is dominated by collisions in the central intermeshing region, there is little difference between the particle sizes that fit through this outer gap (200 µm, 400 µm) and those that are too large (1500 µm particles excluded) to pass through. This suggests that the screw–barrel clearance is not a significant concern when choosing particle size.

Finally, it is worth considering the qualitative particle dynamics when investigating particle size effects. Figure 11 compares the snapshots at the same time instant for all simulations. For the 1500 µm particles, there is no clear or obvious pattern in the particle dynamics due to the low number of particles, with just under 1200 existing at this particle size. However, a flow pattern starts to appear when the particle size is decreased to 1000 µm (approximately 4100 particles). At 800 µm and 600 µm, this “*x-shaped*” crossover pattern becomes increasingly well defined. Further reductions in particle size do not significantly enhance this flow pattern. This was checked for particles as small as 100 µm (Figure 11g). The 400 µm reference case is a good compromise between computational efficiency and capturing the particle dynamics of the system, and 400 µm has been selected as the reference size for the study. Omitted from the comparison in Figure 10 were 100 µm particles due to the large number of particles (approx. 4.1 M in total) and limited simulation time limiting the amount of available data.

## 3. Sensitivity Study of DEM Input Parameters

The results of a sensitivity study on the mean particle diameter are presented in Figure 10 and Figure 11. For the remaining parameters, the sensitivity study employs a univariate approach to quantify the effect of a DEM parameter relative to the reference case defined in Table 3. In cases where the reference value is an intermediate value, both extreme high and low values were tested; when the reference value is already an extreme value, an opposite extreme value was tested without an intermediate value. Variations from the reference case are given in Table 4.

For the cohesive case, the contact plasticity ratio, tangential stiffness multiplier (ζ_tm_), slope exponent (*n*) and tension exponent (*x*) were fixed at 0.8, 0.667, 1.5 and 20, respectively. The contact plasticity ratio was arbitrarily chosen as a value suitable for a highly compressible cohesive powder and the adhesion energy is chosen as 15 J/m^2^ and *f*_0_ = −0.001 N. Both *n* and ζ_tm_ are consistent with the non-linear Hertz model used for the cohesionless simulations.

### 3.1. Particle Size Distribution

**Figure 12:**
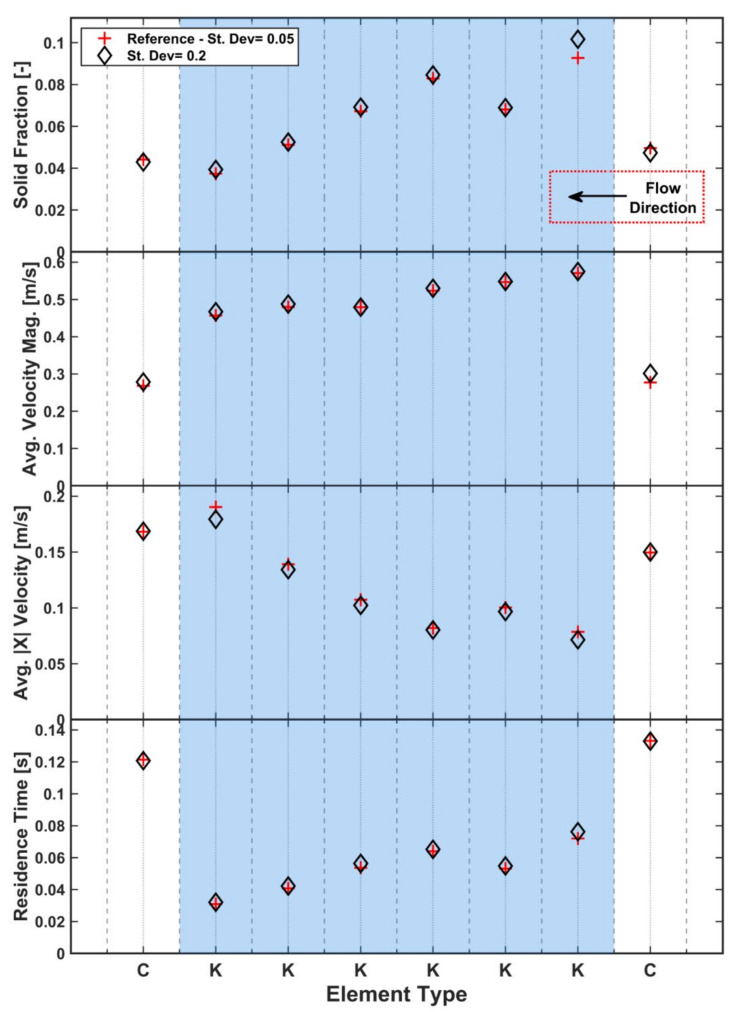
Effect of particle size distribution on solid fraction, velocity and residence time.

Two different size distributions with the same mean (400 µm) were considered, with both distributions truncated at 200 µm and 600 µm. Adopting the broader distribution yields far more large and small particles in a simulation than the narrower distribution. Figure 12 shows the effect of the size distribution on the various measured quantities for each element. In general, the spread of the size distribution has little effect. The exception is the first kneading element at which there is approximately a 10% difference in the solid fractions. The transition from the conveying elements to the first kneading element is a highly chaotic region, with much backflow from other kneading elements meeting the forward flow from the conveying. This can lead to large fluctuations at this element when there is a large variation in particle sizes. A broader size distribution leads to a slightly reduced average longitudinal velocity in the kneading elements, while the conveying elements, which are typically plug flow due to the geometric constraint, have almost identical velocities despite the variation in size distribution. This correlates well with the solid fraction: the slightly reduced longitudinal velocity increases the solid fraction and, hence, the observed residence times at the same mass flow rate. The average velocity magnitude, which includes the in-plane particle movement, remains almost identical for both distributions.

### 3.2. Coefficient of Restitution

**Figure 13:**
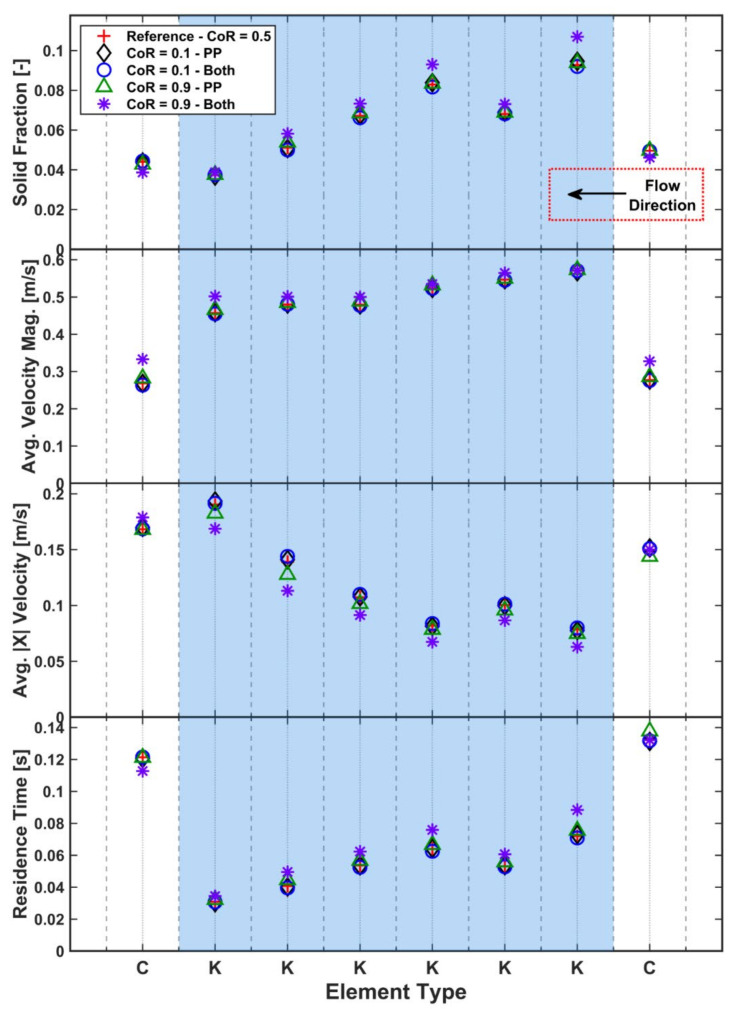
Effect of coefficient of restitution on solid fraction, velocity and residence time.

The effect of damping in the simulations has been considered in terms of both particle–particle and particle–geometry collisions to establish which has the greater influence on the results. Figure 13 shows the effect of damping on the various quantities computed. Reducing the coefficient of restitution, χ, (increasing damping) from the reference value of 0.5 to 0.1 for either interparticle or particle–geometry contacts has little effect on the averaged solid fraction. Increasing χ to 0.9 for interparticle contacts similarly has little effect.

However, increasing χ for both the particle–geometry contacts and the interparticle contacts leads to a significant effect on the average solid fraction for both kneading and conveying elements. Low particle–geometry damping leads to more chaotic behaviour as less energy is dissipated. The increased chaotic behaviour appears to be preventing particles from freely moving axially through the granulator, shown by the reduced axial velocity for kneading elements in Figure 13, which in turn leads to higher residence times for the kneading elements. While the longitudinal velocity is decreased when the particle–geometry coefficient of restitution is increased, the opposite trend is seen in the average velocity magnitude. This shows that there is significantly increased in-plane velocities, which leads to the larger velocity magnitude. Provided that a sensible value is chosen, the TSG model is relatively insensitive to the amount of damping between particles or geometries, with the particle–geometry coefficient of restitution being the more influential. This effect is likely to be relative to the screw speed, with higher screw speeds likely to exaggerate the effect.

### 3.3. Static & Rolling Friction Coefficients

**Figure 14:**
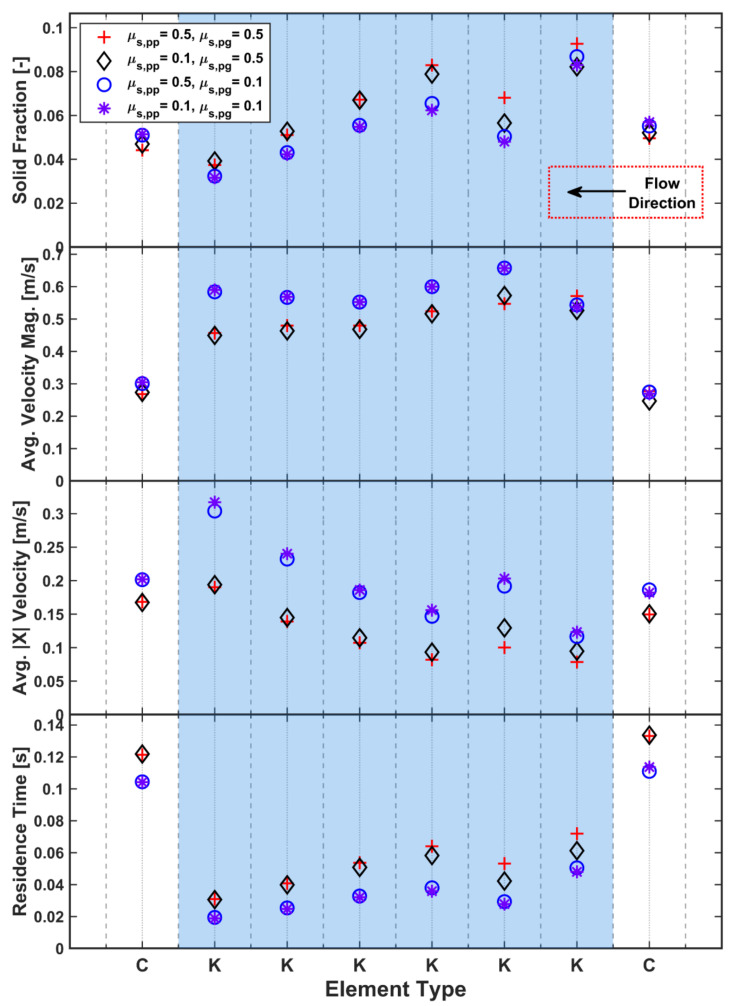
Effect of static friction coefficient on solid fraction, velocity and residence time.

The static friction coefficient, μ_s_, is often a key parameter as it makes a significant contribution to the generation of shear strength in DEM simulations. The reference case used a relatively high value of μ_s_ = 0.5. Increasing μ_s_ beyond this value will not cause very large changes as the effect of particle friction tends to saturate. This limited study investigated the effect of friction by decreasing μ_s_ to 0.1 for both particle–particle (μ_s,pp_) and particle–geometry (μ_s,pg_) contacts, individually and in tandem. When the averaged solid fraction for the four cases is studied (Figure 14), the results fall into two distinct groups distinguished by the μ_s,pg_ values. A lower friction coefficient leads to significantly lower solid fractions across all elements, particularly for the kneading elements. The trend is less well defined for the first and second kneading elements due to the nearby transition from conveying to kneading. The division into two groups defined by μ_s,pg_ is even more apparent in the longitudinal velocity (also in Figure 14). Larger μ_s,pg_ leads to much lower longitudinal velocities as the increased friction leads to increased shear along the barrel surface, retarding flow. This also increases the residence time for the higher μ_s,pg_. As particles’ longitudinal velocity reduce and they spend longer on each element, the in-plane velocity also increases due to the longer time spent being rotated by the element. This leads to an increase in the velocity magnitude, especially for the kneading element at which the longitudinal velocity is the lowest. In general, μ_s,pp_ has minimal influence in the TSG, which appears to be a system dominated by geometry interactions and geometry dynamics.

Rolling friction is a commonly used DEM approach to incorporate the effect of particle shape without the computational expense of simulating non-spherical particles. It works by applying an additional torque to resist particle rolling. The reference case used a minimal value of 0.01 as the rolling friction coefficient. In the same manner as for static friction, the opposite extreme is explored for particle–particle (μ_r,pp_) and particle–geometry (μ_r,pg_) coefficients to assess the effect on the TSG model.

**Figure 15:**
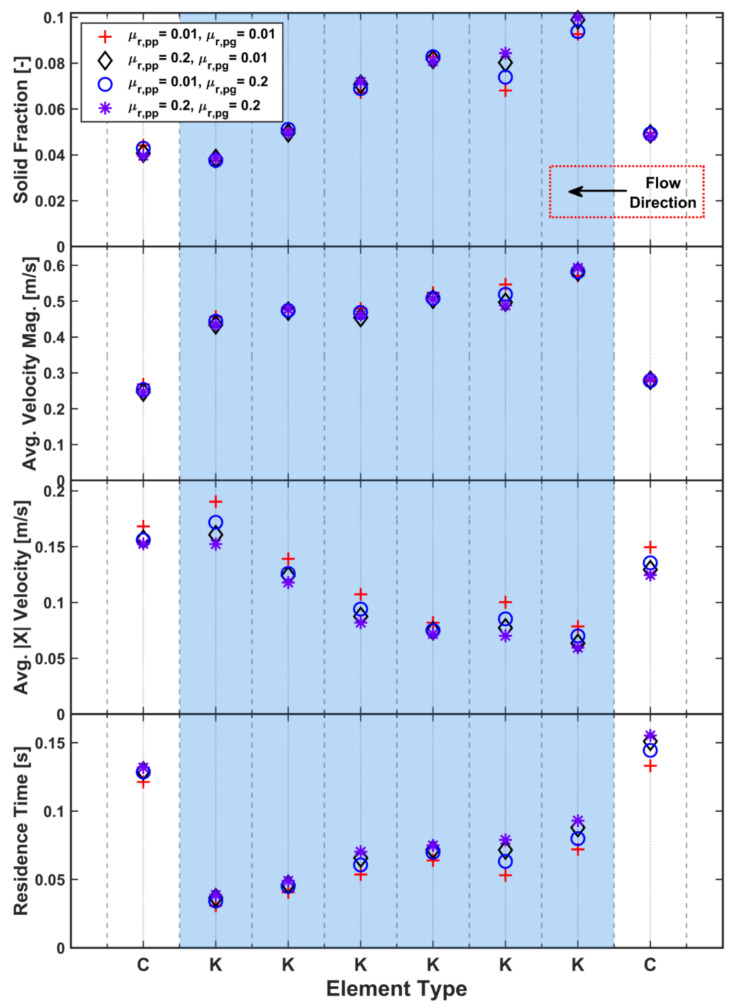
Effect of rolling friction coefficient on solid fraction, velocity and residence time.

Figure 15 shows that rolling friction has a minimal effect on the average solid fraction on most elements, except for the first and second kneading elements. More particles are held up on these elements when either rolling friction coefficient increases. Increasing rolling friction leads to reduced longitudinal velocities across all elements. Increasing μ_r,pp_ and μ_r,pg_ has a similar effect, although the former seems to have a greater significance: the longitudinal velocity reduced more for particle–particle contacts than for particle–geometry contacts. The combination of both leads to the largest reduction in velocity. The average residence time (Figure 15) is strongly linked to the average longitudinal velocity and displays the same trends, with μ_r,pp_ having the most significant effect.

### 3.4. Effect of Cohesion

**Figure 16:**
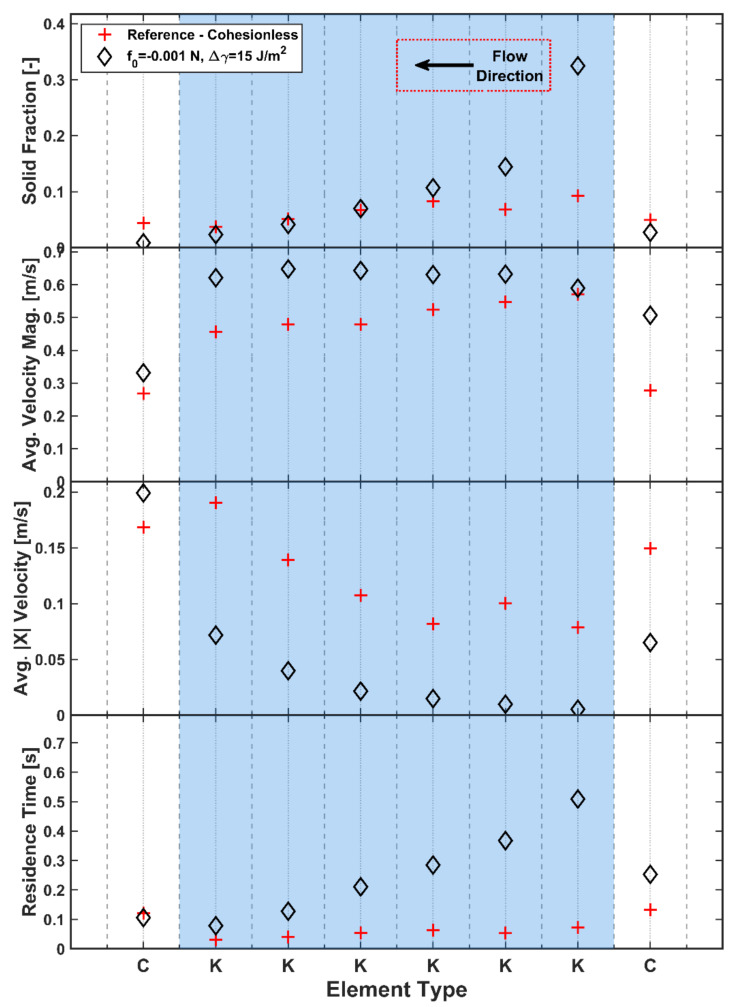
Effect of level of cohesion on solid fraction, velocity and residence time.

The introduction of cohesion into the system is required to capture the behaviour of the real material, which will have significant levels of cohesion arising from the added liquid binder. The additional cohesion leads to dramatically different behaviour (Figure 16) in comparison to the previously explored cohesionless material as agglomerates begin to form in the granulator. There is a significant increase in the measured solid fraction for the first two kneading elements; thereafter, the solid fraction reduces to be much closer to the cohesionless reference case. The conveying elements show a significant decrease in solid fraction, which is a consequence of the mass balance requirement of a periodic system. With the initial mass fixed (based on the cohesionless steady-state mass), a significant increase in solid fraction in one area must be compensated by decreases in solid fraction elsewhere. In this case, cohesion has significantly decreased the mass flow rate on the conveying element from the expected 14.4 kg/h.

The average longitudinal velocities also decrease when cohesion is introduced, as it is now more difficult for particles to flow forward individually. The effect of cohesion on the longitudinal velocity may also be enhanced as a side-effect of the periodic system. As the mass balance requirements have led to a drop in the mass flow rate on the conveying input section, there is now less forward momentum pushing particles through the kneading zone, which leads to the significant drop in longitudinal velocity. As a result of the reduction in longitudinal velocities, the average residence times (Figure 16) increase significantly with increasing cohesion.

In terms of solid distribution on the elements, which is displayed in Figure 7 for the reference case of 400 µm cohesionless particles, a preliminary study on 200 µm particles (not included in this paper) indicates that, for the conveying elements, the introduction of cohesion reduces the amount of material transported along the bottom of the barrel. With cohesion, a greater proportion of the material is caught on the intermeshing region between the screws due to the formation of agglomerates. The kneading elements have the same flow patterns with and without cohesion. However, the solid fraction increases on average across the section, particularly at the outer extremes of the element and at the intermeshing zone due to the greater amount of material build-up on these elements.

**Figure 17:**
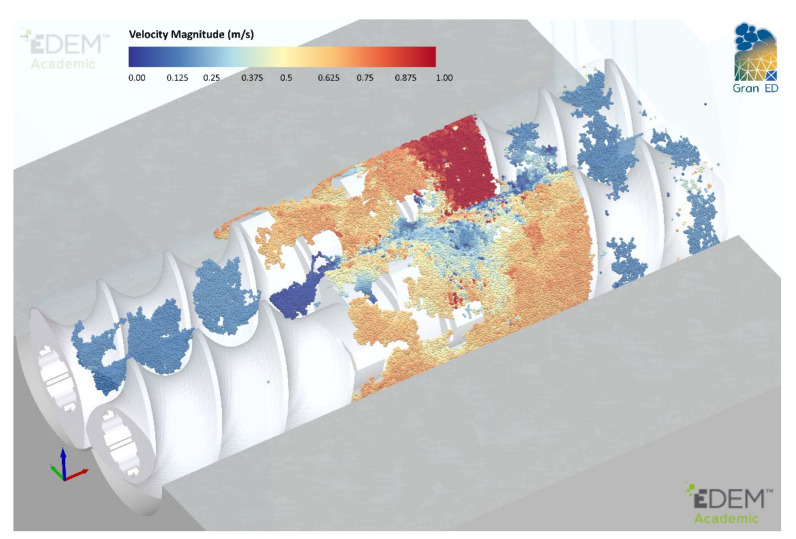
Agglomerate formation in the non-periodic reduced domain model (direction of flow: right to left).

As previously mentioned, in order to study the formation of granules, a non-periodic reduced domain model is required to prevent recycling of formed granules. This process is shown in Figure 17 where virgin material is generated at the rightmost side of the domain. In this model some small agglomerates are formed on the intermeshing region of the screws and transported towards the kneading elements. The kneading elements create a build-up of material and lead to significant consolidation in this zone, helping to create dense agglomerates. As these dense agglomerates leave the kneading zone, there is a slight chopping action of the screw element in the intermeshing region, which helps break up the dense agglomerates and create some independent agglomerates. These agglomerates are still quite large at this point and would most likely be significantly reduced in size by the chopping elements (not included in this reduced domain model) normally included at the TSG outlet. The inclusion of cohesion in the model is highly significant in capturing the natural formation of granules in a TSG.

Capturing the formation of agglomerates within the TSG is a key aspect of the model, and this is accomplished though the inclusion of the cohesive forces, which represent the liquid binder in the real granulator. No changes in primary particle sizes occur in the model; instead, primary particles agglomerate because of the cohesive forces.

## 4. Discussion

In this study, the reference case was the full-scale, cohesionless system, which had a mass flow rate set at 14.4 kg/h. The initial mass of the periodic system was calculated from the steady-state operation of the full-size model. The computationally efficient periodic system is then used extensively to assess how the DEM input parameters would influence the behaviour in the granulator. The results in Section 3 have considered how the particle dynamics in the various elements were affected in terms of velocity, solid fraction and residence time. However, due to the periodic system having a fixed mass, it is possible to measure the change in mass flow rate on the conveying elements at the inlet and outlet of the periodic domain. Any significant changes to the bulk behaviour will result in variations in the observed mass flow rates and a difference from the full-scale model value, which can be used to assess the magnitude of a parameter effect. The larger the difference is, the more influential that parameter is.

**Figure 18:**
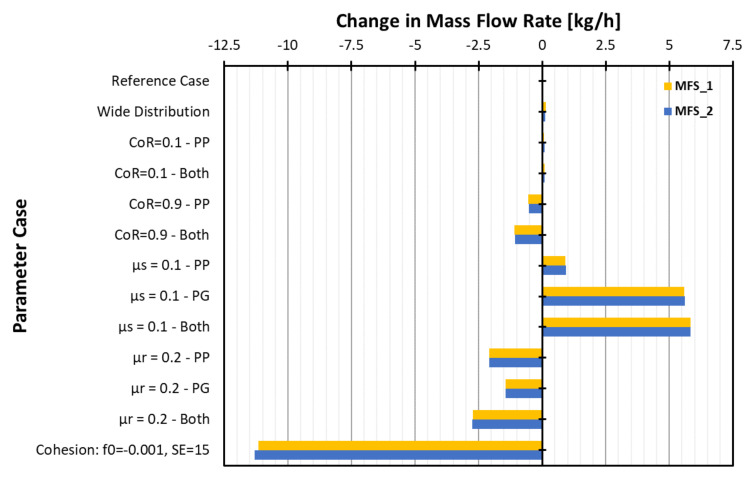
Observed changes in mass flow rate in the periodic system.

To consider this effect and the sensitivity of the mass flow rate to the parameters, the mass flow rates were calculated at sensors (MFS_1 and MFS_2) at each end of the periodic system and plotted for each case in Figure 18. Steady state exists in the system when the averaged mass flow rate is approximately equal for both the inlet and outlet sensor.

For a changing size distribution and low restitution, there is almost no change from the expected 14.4–14.6 kg/h mass flow rate. The decrease in damping at χ = 0.9 leads to slightly higher solid fractions being observed for the kneading elements, which had the effect of slightly reducing the mass flow to 13.7–14.1 kg/h.

The reduction in static friction (μ_s_) led to reduced solid fractions, which manifested as a significant increase in the mass flow rate of approximately 5 kg/h to just over 20 kg/h. Increasing rolling friction, μ_r_, led to an increase in solid fraction for some elements, which led to a reduction in the mass flow rate to approximately 12–13.3 kg/h depending on the combination of parameters used. The effect of cohesion is the most dramatic, with the mass flow rates reduced by just over 10 kg/h for the cohesive case. This is due to the significant agglomeration and build-up of solids on the kneading elements, which reduces the circulating mass in the periodic system. The dominant effect of cohesion explains why it was considered separately from other parameters, allowing the relative importance of each to be quantified without being outweighed by the more dominant parameters.

## 5. Conclusions

Discrete Element Method (DEM) simulations were used in this study of the particle dynamics that occur in a twin-screw granulator comprising both conveying and kneading elements. Both full-scale and reduced periodic models were used.

The DEM simulations for a cohesionless system provide a mean residence time—a key characteristic of a TSG—that is in line with expectations when compared to experimental results with various different granulators and cohesive materials. The residence time increased, as expected, when an adhesive, elasto-plastic contact model was used to capture the effect of liquid binder and the resulting agglomeration in the granulator.

The significant computational expense of the full-scale simulations was reduced through (carefully defined) periodic simulations. Results show that the residence time distributions across elements in a periodic simulation are almost identical to their counterparts in the full-scale simulation. These reduced domain models allow the key particle dynamics to be captured at a much lower computational cost, making larger studies more feasible in the future. These models, combined with a DEM contact model that includes cohesion, are an effective way to simulate and investigate the wet granulation process.

A sensitivity study showed that the level of cohesion is a key determinant of granule size and must be carefully calibrated using experiments. An important finding is that the size of the fundamental particles in the simulation may be larger than in reality while still capturing the correct dynamics for the TSG. The results suggest the existence of a critical ratio of particle diameter to the screw void space (effectively the difference between the screw’s root and outer radii). Once the particle size is below a certain threshold, the velocity, solid fraction and residence time are largely unaffected by particle size, with the exception of the first kneading element where there is the transition from the conveying elements. This is a highly chaotic region that appears to be heavily influenced by particle size. This effect was also noted when a broader particle size distribution was used; the increased numbers of larger particles lead to slightly increased solid fractions and residence times on the first kneading element only. The effect of the screw–barrel clearance is also found to be negligible with particles that are both smaller and larger than this critical size behaving the same in most locations in the granulator.

The effect of restitution coefficient (damping) was relatively insignificant for materials not considered to be elastic materials. The particle–geometry damping is more influential than the particle–particle damping, which is a feature of a system where geometries are moving at very high speeds, leading to high relative impact velocities. Similar trends were seen for static friction: reducing the particle–geometry static friction leads to significant changes in the solid fraction and residence times. Rolling friction has a lesser effect than static friction, in general. Increased cohesion leads to increased solid fractions and residence times on kneading elements. The results suggest that the TSG is a system that is dominated by geometry interactions, with velocities very much controlled by the screw speed and pitch.

## Figures and Tables

**Table 1:** Key ConsiGma 1/25 Dimensions.

Property	Value
Barrel Diameter, D [mm]	25
Channels [-]	2
Channel Depth [mm]	2.58
Centre line Distance [mm]	19.31
Barrel–Screw Clearance [mm]	0.440
Screw–Screw Clearance [mm]	0.175
Area of Double Intermeshed Barrels [mm^2^]	920.0
Free Barrel Area [mm^2^]	435.26
Free Barrel Fraction	0.4731

**Table 2:** Key ConsiGma Screw Properties.

Property	Value
Screw Radius—Outer, R [mm]	12.06
Screw Radius—Inner, r [mm]	6.90
Screw Diameter Ratio	1.75
Screw Transition Radius, R + r [mm]	18.96
Screw Length, L [mm]	500
Screw Lead, S [mm]	25
Screw Section Perimeter [mm]	59.56
Screw Section Area [mm^2^]	242.37
Screw Transition Angle	38.15°
Screw Tip Angle	13.72°

**Table 3:** Reference values of the DEM input parameters.

Property	Particles	Geometry
Poisson’s ratio, ν	0.25	0.25
Shear modulus, G (Pa)	1.7 × 10^7^	1 × 10^8^
Coefficient of restitution, χ	0.5	0.5
Static friction, µ_s_	0.5	0.5
Rolling friction, µ_r_	0.01	0.01
Density (kg/m^3^)	1263	7850
Mean particle radius (m)	0.0002	
Standard deviation	0.05	
Normalised truncation limits	(0.5, 1.5)	
Gravitational acceleration (m/s^2^)	9.81	

**Table 4:** Variations from the reference case defined in Table 3, where χ, µ_s_ and µ_r_ apply to both particle–particle and particle–geometry interactions.

	Reference	Variations
Coefficient of restitution, χ	0.5	0.1, 0.9
Static friction, µ_s_	0.5	0.1
Rolling friction, µ_r_	0.01	0.2
Relative Standard Deviation	0.05	0.2
Cohesion	Cohesionless	High (γ = 15 J/m^2^, *f*_0_ = −0.001 N)

## Data Availability

The data presented in this study are openly available on Edinburgh DataShare at https://doi.org/10.7488/ds/3251. Data citation: Morrissey, John P; Hanley, Kevin; Ooi, Jin Y. (2021). Data from “Conceptualisation of an efficient particle-based simulation of a twin-screw granulator”, [dataset]. University of Edinburgh. School of Engineering. Institute for Infrastructure & Environment. https://doi.org/10.7488/ds/3251.
